# APHA session features emerging infections.

**DOI:** 10.3201/eid0104.950415

**Published:** 1995

**Authors:** M S Favero


instructors included statisticians and epidemiologists from the Division of Parasitic Diseases, National Center for Infectious Diseases, Centers for Disease Control and Prevention in Atlanta, Georgia, and in Guatemala City, Guatemala, and staff from the National Aeronautics and Space Administration, Center for Health Application of Aerospace Related Technologies, Ames Research Center, Sunnyvale, California.

Objectives of the training included the following: mastery of the principles and general concepts of all GIS systems; use of Atlas GIS/DOS to associate map files with databases to produce thematic maps, manipulate various layers (rivers, highways, village locations) of the map files to produce customized maps, create buffers around geographic features, and use them in simple analyses; designing georeferenced data files that can be read by the GIS; digitizing paper maps to acquire new data for building a GIS; use of GPS to obtain latitudes, longitudes, and elevations of villages and other major landmarks and to use this information in the GIS; and mastery of importing/exporting databases and map files.

The course was designed to enable participants to set up and use a GIS for research, planning, or operational purposes. Participating were institutions from Mexico (two teams), Colombia (two), Puerto Rico, Costa Rica, Venezuela, Guatemala (two), Ecuador, and Brazil. Each team came to the course with ideas, maps, and data pertaining to an existing project that would be continued at their home institution. Student project areas included onchocerciasis, malaria, water sanitation, leishmaniasis, and public health and natural resource utilization/preservation. The students were taught digitizing and were asked to use Guinea worm surveillance data to create their own GIS.

A full day was devoted to geographic analyses. Topics covered included aggregating data from one geographic layer to another, combining geographic features with common database values, and combining selected features to form new map layers. A workshop on remote sensing, GIS, and image classification explained that satellite imagery and remotely sensed data are obtained by measuring reflectance on seven spectral frequencies and that ground cover can be partially deduced by the amount of reflectance at each band. Field exercises to practice GPS use in the Lake Atitlan area followed. Another workshop covered advanced digitizing and gave each team a good start on the digitizing part of their projects. Individual instructions were given on how to import map files from other GIS programs into Atlas GIS. Lastly, the Guatemalan onchocerciasis GIS system was presented as a case study.

In addition to the 2 weeks of training, each participating institution received a copy of all lecture notes, the critical hardware needed to continue the project at home, and the following software, complete with documentation: Atlas GIS/DOS, Import-Export, and Arcview 2. An ongoing Internet-based discussion group for class organizers and participants is providing a forum for dialogue and monitoring of participants' progress.

**Allen W. Hightower**
**Robert E. Klein**
National Center for Infectious Diseases
Centers for Disease Control and Prevention
Atlanta, Georgia, USA, and Guatemala City, Guatemala

### APHA Session Features Emerging Infections

Emerging and reemerging infections will be the featured topic of a two-part session at the annual meeting of the American Public Health Association, October 29-November 2, in San Diego, California.

The session, titled "Emerging Infections: Solving the Mysteries in the Field and Laboratory," will focus on the worldwide impact of new and reemerging infections from both an epidemiologic and a laboratory perspective.

Eight speakers from national and international health organizations will discuss the following aspects of the public health threat of these diseases: public health strategies for controlling infectious diseases; social, geographic, ecologic, and environmental factors that have allowed these diseases to spread; the growing threat of antimicrobial resistance; the increased need for accurate and meaningful disease surveillance; and the challenge to apply the latest laboratory technology to rapidly detect and characterize new infectious agents.

**Martin S. Favero**
National Center for Infectious Diseases
Centers for Disease Control and Prevention
Atlanta, Georgia, USA

